# Use of an online e-learning module to standardise the assessment and reporting of a subjective endpoint in a multicentre rct

**DOI:** 10.1186/1745-6215-14-S1-P64

**Published:** 2013-11-29

**Authors:** Thomas Pinkney, David Bartlett, Adrian Gheorghe, George Dowswell, Dion Morton, Melanie Calvert

**Affiliations:** 1 West Midlands Research Collaborative, Birmingham, UK; 2 Primary Care Clinical Sciences, University of Birmingham, Birmingham, UK; 3 Academic Department of Surgery, University of Birmingham, Birmingham, UK

## Background

Multicentre trials employing a subjective primary outcome measure have the potential to suffer from significant inter-rater variability which may compromise the validity of findings. The primary outcome for the ROSSINI Trial was surgical site infection. We designed a bespoke online training module with embedded quiz to ensure standardisation and reproducibility of wound assessment.

## Methods

We designed an online e-learning module comprising an educational review of surgical site infection signs and symptoms, as well as a quiz with high quality digital photos of surgical wounds to ensure optimal and standardised diagnosis. Automated feedback was given instantly to maximise and reinforce learning.

## Results

The e-learning module and quiz was completed by the majority of local investigators at the 21 sites that opened for the ROSSINI trial. A total of 769 patients were recruited to the trial and each patient underwent at least two blinded wound reviews as part of the primary outcome assessment.

## Conclusion

The online learning resource provided effective training and accreditation to wound reviewers at multiple sites in a convenient and reproducible manner. This adjunct to our trial served to standardise outcome assessment and hopefully reduced inter-observer variability.

